# Loss of neurotrophin-3 from smooth muscle disrupts vagal gastrointestinal afferent signaling and satiation

**DOI:** 10.1152/ajpregu.00337.2013

**Published:** 2013-09-25

**Authors:** Edward A. Fox, Jessica E. Biddinger, Zachary C. Baquet, Kevin R. Jones, Jennifer McAdams

**Affiliations:** ^1^Behavioral Neurogenetics Laboratory, Department of Psychological Sciences, Purdue University, West Lafayette, Indiana; and; ^2^Department of Molecular, Cellular & Developmental Biology, University of Colorado, Boulder, Colorado

**Keywords:** conditional knockout, food intake, meal patterns, meal microstructure, vagus nerve

## Abstract

A large proportion of vagal afferents are dependent on neurotrophin-3 (NT-3) for survival. NT-3 is expressed in developing gastrointestinal (GI) smooth muscle, a tissue densely innervated by vagal mechanoreceptors, and thus could regulate their survival. We genetically ablated *NT-3* from developing GI smooth muscle and examined the pattern of loss of NT-3 expression in the GI tract and whether this loss altered vagal afferent signaling or feeding behavior. Meal-induced c-Fos activation was reduced in the solitary tract nucleus and area postrema in mice with a smooth muscle-specific *NT-3* knockout (*SM-NT-3*^*KO*^) compared with controls, suggesting a decrease in vagal afferent signaling. Daily food intake and body weight of *SM-NT-3*^*KO*^ mice and controls were similar. Meal pattern analysis revealed that mutants, however, had increases in average and total daily meal duration compared with controls. Mutants maintained normal meal size by decreasing eating rate compared with controls. Although microstructural analysis did not reveal a decrease in the rate of decay of eating in *SM-NT-3*^*KO*^ mice, they ate continuously during the 30-min meal, whereas controls terminated feeding after 22 min. This led to a 74% increase in first daily meal size of *SM-NT-3*^*KO*^ mice compared with controls. The increases in meal duration and first meal size of *SM-NT-3*^*KO*^ mice are consistent with reduced satiation signaling by vagal afferents. This is the first demonstration of a role for GI NT-3 in short-term controls of feeding, most likely involving effects on development of vagal GI afferents that regulate satiation.

neurotrophin-3 (nt-3) is a member of the mammalian neurotrophin family of secreted proteins. The high-affinity receptor for NT-3 is the receptor tyrosine kinase C, trkC (52), although NT-3 can also activate trkA or trkB (14, 30). Additionally, p75 can be independently activated by each neurotrophin, and it may partner with any one of the neurotrophin trk receptors to mediate specific neurotrophin responses (10). NT-3 supports the development of a wide range of peripheral sensory systems. For example, NT-3 deficiency is associated with the absence of auditory afferents that arise from the spiral ganglion, and somatosensory afferents that arise from the dorsal root ganglia (DRG). These include proprioceptive afferents and their peripheral sense organs (muscle spindles, Golgi tendon organs), D-hair afferent mechanoreceptors, and slowly adapting cutaneous mechanoreceptors and their associated Merkel cells, which are lost postnatally (27, 29, 39, 53). The loss of these somatosensory pathways, in particular those contributing to proprioception, are thought to contribute to the abnormal movements and difficulty feeding observed in *NT-3* knockout (KO) mice, which die by 3 wk of age. Also, partial NT-3 loss (in heterozygotes, +/−) results in partial loss of D-hair afferents and slowly adapting mechanoreceptors, but those that survive have normal function (1). In contrast, cutaneous overexpression of NT-3 leads to increased innervation of touch domes and hair follicles, including increased number of both Merkel cells and the DRG neuron cell bodies from which these afferents originate (2).

NT-3, acting in part through activation of the receptor trkC, is essential for the survival of a large proportion of vagal sensory neurons: *NT-3* and *trkC* homozygous mutants have 34–47% and 14% loss of neurons from the nodose-petrosal ganglion complex, respectively (27, 29, 58, 84). Although the full range of peripheral targets of these NT-3-dependent vagal sensory neurons remains unknown, vagal afferents that innervate the esophagus appear to be among them. In particular, intraganglionic laminar ending (IGLE)-type vagal mechanoreceptors that predominate in the esophageal muscle wall are reduced by 65 and 40% in *NT-3* and *trkC* heterozygous mutants, respectively (71).

The support of vagal sensory neuron survival by NT-3 could be mediated by its expression in the developing nodose ganglion (28), the brain stem targets of nodose afferents, or by NT-3 present in embryonic and early postnatal gastrointestinal (GI) tract tissues innervated by vagal sensory neurons. NT-3 expression in the majority of developing GI organs is largely restricted to smooth muscle cells comprising the outer layers of the developing stomach, cecum, small and large intestines, and the walls of blood vessels that supply the GI tract (33, 36). However, each organ exhibits a unique expression pattern. These patterns differ in terms of which muscle layer exhibited NT-3 expression, whether any additional tissues exhibited expression, and the temporal pattern of expression. For example, NT-3 is expressed in the developing lamina propria in the antrum and corpus of the stomach, but not in the lamina propria of the forestomach or intestines. Also, NT-3 expression occurs at high levels in GI mesenchyme (tissue layer from which GI smooth muscle is derived) by embryonic day (E)12. In contrast, NT-3 expression in vascular smooth muscle is first observed at E15 at low levels that increased gradually with age. Consistent with a possible contribution of GI NT-3 to regulation of vagal afferent development, both trkC and trkB are expressed by nodose ganglion neurons from E13 to E18 (28, 48, 49). Furthermore, vagal innervation of the gut begins developing over the same timeframe as expression of neurotrophins and their receptors. For example, developing vagal axons begin to populate the upper GI tract at E12, and vagal mechanoreceptors begin to form in the smooth muscle wall at E16 (65).

In addition to the possible contribution of embryonic and early postnatal GI NT-3 expression to vagal sensory neuron survival, this source of NT-3 could have other effects on development of vagal afferents. Additional actions of NT-3 have been characterized in the sensory and sympathetic nervous systems, including roles in neuronal differentiation (26), axon growth (41, 86), and nerve terminal formation, including terminal size and structure (56, 87), degree of contact of nerve terminals with their accessory cells, and survival of their accessory cells (2).

Based on the effects of *NT-3* KO on vagal sensory neuron survival and other aspects of development of sensory and autonomic systems described above, we predicted that the specific loss of NT-3 from GI smooth muscle would disrupt development of vagal sensory innervation of the GI tract and consequently reduce vagal signaling to the brain. To investigate this hypothesis, mice with reduced NT-3 levels in GI smooth muscle were generated using a conditional gene targeting strategy, and vagal signaling from gut to brain in these mice was assessed by quantifying meal-induced activation of neurons in the dorsal vagal complex (DVC) and quantifying relevant meal pattern and microstructure parameters.

## MATERIALS AND METHODS

### Animals

#### Animals.

*SM22α*^*cre*^ [Ref. 46; also referred to as *transgelin*^*cre*^; Tg(*Tagln-cre*)1Her/J; cat. no. 004746, JAX Laboratories, Bar Harbor, ME], *NT-3*^*neo/+*^ (Ref. 85; obtained from Lino Tessarollo), *NT-3*^*+/lox*^ (Ref. 4; obtained from Guoping Fan), and *NT-3*^*+/lox-lacZ*^ (described below) mice were maintained at 23°C on a 12:12-h (14:10 h for breeding) light-dark schedule, lights on at 0500, with ad libitum access to tap water and Laboratory Rodent Diet 5001 (PMI Nutrition International, St. Louis, MO). All procedures were conducted in accordance with the *NIH Guide for the Care and Use of Laboratory Animals* (8th ed., The National Academic Press, Washington, DC) and American Association for Accreditation of Laboratory Animal Care guidelines and were approved by the Purdue University Animal Care and Use Committee.

#### Rationale for use of SM22α^cre^ mice to target NT-3 KO to smooth muscle.

The *SM22α*^*cre*^ mouse strain was utilized because it meets several criteria required for testing the role of GI NT-3 in vagal afferent development and function, as described previously for GI brain-derived neurotrophic factor (BDNF) (34). First, expression of Cre recombinase in *SM22α*^*cre*^ mice is largely restricted to smooth muscle, the main GI tissue that expresses NT-3 (33, 34, 36, 57). Second, tests with a Cre-dependent reporter strain provided evidence of high recombination efficiency in smooth muscle (34, 57). Third, we previously characterized *SM22α*^*cre*^-mediated recombination at several developmental stages using *Rosa26* reporter mice. We and others found that *SM22α*^*cre*^ mice produce sufficient Cre expression to drive recombination in smooth muscle of the GI wall and associated blood vessels at ages when vagal GI afferents are entering the gut and developing there (34, 65, 69, 74, 94). Fourth, global *NT-3* KO mice exhibit abnormal movements and postures and die shortly after birth (85). A smooth muscle-specific KO of *NT-3* produced using *SM22α*^*cre*^ mice, however, was predicted to be viable and exhibit normal movements and postures, since the global loss of NT-3 that leads to these symptoms will be greatly reduced, being largely restricted to smooth muscle. Finally, *SM22α*^*cre*^ mice have proven to be the most successful smooth muscle Cre driver strain, having been utilized by several different investigators to eliminate numerous genes of interest from smooth muscle (11, 34, 40, 44, 55, 62, 69, 72, 80, 83, 88).

#### Generation of smooth muscle-specific NT-3 KO mice.

To restrict the *NT-3* KO mainly to smooth muscle, *SM22α*^*cre*^ and *NT-3*^*neo/+*^ mice were crossed to generate *SM22α*^*cre/+*^;*NT-3*^*neo/+*^ mice, which were mated to *NT-3*^*+/lox*^ mice to obtain *SM22α*^*cre/+*^;*NT-3*^*+/lox*^ and *SM22α*^*cre/+*^;*NT-3*^*neo/lox*^ mice. The latter are referred to as “*SM22α-NT-3*^*KO*^” mice. Thus *SM22α-NT-3*^*KO*^ mice were generated by combining three different mutations in individual mice, with each mutation contributing to reducing NT-3 expression in a different manner. These mice had a global KO of one *NT-3* allele (*NT-3*^*neo/+*^ mutation). This *NT-3* mutation resulted from the insertion of the neomycin-resistance gene with the phosphoglycerate kinase (PGK) 1 promoter and the bovine growth hormone polyadenylation sequence in the middle of exon II of the *NT-3* gene, which codes for the entire NT-3 polypeptide (85). *SM22α-NT-3*^*KO*^ mice also had loss of the other *NT-3* allele in a high percentage of GI smooth muscle cells: recombination efficiency of the *SM22α*^*cre*^ transgene in this tissue is high (see results section and Refs. 34, 57; recombination was due to interaction of Cre recombinase expressed by the *SM22α*^*cre/+*^ transgene with its *loxP* target sites at the *NT-3* locus). This recombination resulted in removal of most of exon II of the *NT-3* gene (4). We utilized this conditional KO strategy because it improves the efficiency of the *NT-3* KO, and it avoids potential mitotic recombination that can occur in mice with *loxP* sites present on more than one chromosome (76). *SM22α*^*cre/+*^;*NT-3*^*+/lox*^ mice lacked one *NT-3* allele in a high percentage of GI smooth muscle cells and thus harbored a heterozygous smooth muscle-specific *NT-3* KO. *SM22α*^*+/+*^;*NT-3*^*+/lox*^ mice, which have one floxed *NT-3* allele that was not recombined, were used as controls and referred to as “control” mice. These mice were used as controls because they were more abundant than wild types among the offspring that resulted from the breeding strategy employed, and the *loxP* sites inserted into the *NT-3* locus do not appear to alter NT-3 function (4). Offspring genotypes were determined by polymerase chain reaction analysis of DNA extracted from tail tips removed at weaning, or from the embryonic yolk sac or liver.

#### Generation of the NT-3^lox-lacZ^ mouse strain.

**Fig. 1.**
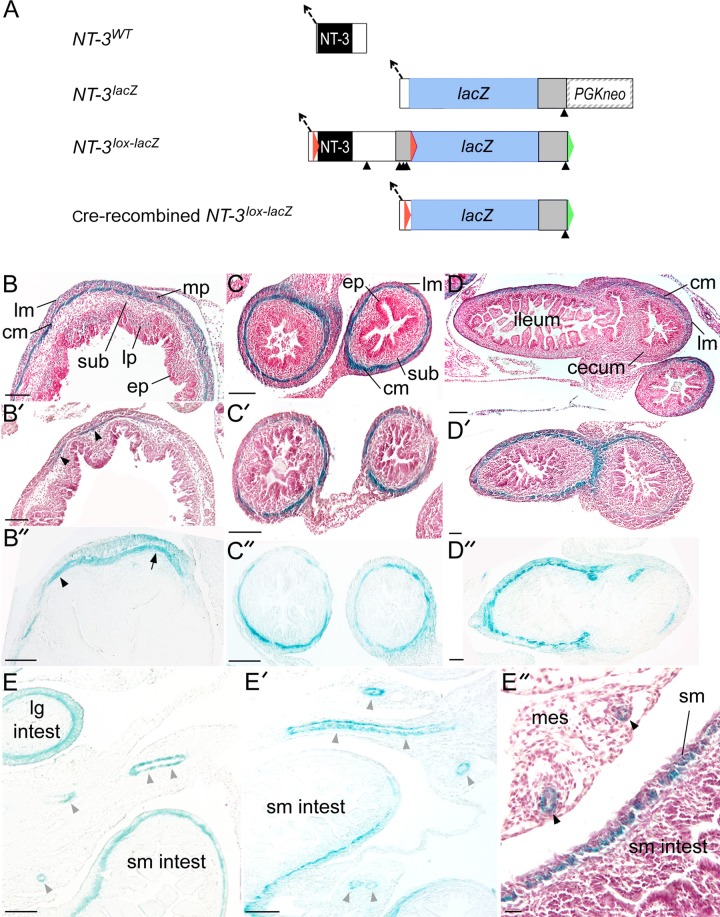
*A*: schematic of the *NT-3*^*lox-lacZ*^ allele structure before and after Cre recombinase-mediated recombination. Schematics describing the structures of the neurotrophin-3 (*NT-3*) wild-type allele (*NT-3*^*WT*^, *first*), the gene-targeted *NT-3*^*lacZ*^ allele (*second*), the gene targeted floxed *NT-3* allele (*NT-3*^*lox-lacZ*^, *third*), and Cre-recombined *NT-3*^*lox-lacZ*^ allele (*fourth*), are shown. For wild-type, the splice acceptor (dashed arrow) at the 5′ end of the *NT-3* coding exon (solid bar, NT-3 protein-coding portion; open bar, noncoding portion) and polyadenylation signal (solid arrowhead below) are indicated. For the *NT-3*^*lacZ*^ allele, the inserted *E. coli lacZ* gene (blue, *lacZ*) with an SV40 intron and polyadenylation signal (shaded box with arrowhead below), followed by the phosphoglycerate kinase (*PGK) neo* selectable marker are shown. For the *NT-3*^*lox-lacZ*^ allele, the inserted *loxP* sequences (red arrowheads), trimerized SV40 polyadenylation signal (shaded box with solid arrowheads below), and *E. coli lacZ* gene (blue, *lacZ*; shaded box and arrowhead, SV40 intron and polyadenylation signal), followed by the *FRT* site remaining after deletion of the *PGKneo* selectable marker are all indicated. *B*–*E*: Cre-mediated recombination in *SM22α*^*cre/+*^;*NT-3*^*+/lox-lacZ*^ and *SM22α-NT-3*^*KO*^ (knockout) mouse embryos, ages embryonic days (E)15–17, represented by β-galactosidase (stained here with X-gal) was largely restricted to sites of NT-3 expression in smooth muscle (sm) in the walls of the stomach, intestine, and blood vessels (bv) supplying the gastrointestinal (GI) tract. Photomicrographs and photomontages of X-gal-stained (blue stain) sections counterstained with neutral red (except panels *B*″, *C*″, *D*″, *E*, and *E*′ were only stained with X-gal) illustrate β-galactosidase expression in the GI tract of *NT-3*^*lacZ*^ embryos, representing NT-3 expression (*B*, *C*, *D*, *E*), and in *NT-3*^*lox-lacZ*^ embryos, representing recombination of *loxP* sites (*B*′, *C*′, *D*′, *E*′, *B*″, *C*″, *D*″, *E*″). NT-3 expression occurred in the smooth muscle of the walls of the stomach (*B*), intestines (*C*), cecum (*D*), and blood vessels supplying the GI tract (*E*). Similarly, Cre-mediated recombination was restricted mainly to smooth muscle of these same tissues (*B*′, *C*′, *D*′, *E*′, *B*″, *C*″, *D*″, *E*″). Note that recombination was only present in a small region of the stomach wall. In *B*′ and *B*″, arrowhead indicates X-gal stain representing Cre-mediated recombination in circular smooth muscle layer only, whereas arrow indicates stain is in both circular and longitudinal smooth muscle layers. Scale bars = 100 μm in all panels except *D*′ and *D*″ = 200 μm. cm, circular muscle; ep, epithelium; lg intest, large intestine; lm, longitudinal muscle; lp, lamina propria; mes, mesentery; mp, myenteric plexus; sm intest, small intestine; sub, submucosa. *D* and *E* were modified from Fox and McAdams (36).

An *NT-3* gene-targeting construct was prepared in which *loxP* sites flank the protein-coding region of the mouse *NT-3* gene (Fig. 1*A*). One *loxP* sequence was inserted in the 5′-untranslated region, 12 base pairs (bp) downstream of the splice acceptor and 29 bp upstream of the translation initiation codon. The other *loxP* site was inserted several hundred bp 3′ of the translation termination codon, downstream of the first polyadenylation signal. The *Escherichia coli lacZ* gene was placed directly downstream of the 3′ *loxP* site, followed by an *FRT*-flanked *PGK* promoter-neomycin phosphotransferase gene. This *FRT-PGKneo-FRT* cassette was previously described in more detail (42). A trimerized SV40 polyadenylation signal (63) was placed immediately 5′ of the 3′ lox site to prevent expression of *lacZ* in the absence of Cre-*loxP* recombination. *Day 3* embryonic stem cells (22) were transfected with the targeting construct, and targeted embryonic stem cell clones were identified and injected into blastocysts, and mice carrying the mutation were derived using standard methods. The resulting strain was mated to the FLP-4917 Flp recombinase-expressing transgenic strain (23), deleting the *PGKneo* by Flp-*FRT* recombination. The *NT-3*^*lox-lacZ*^ mice derived in this manner were backcrossed at least 11 generations to C57BL/6J mice before use in these experiments. The *NT-3*^*lox-lacZ*^ allele is hypomorphic, with reduced NT-3 mRNA and protein accumulation in brain and heart, and homozygous, but not heterozygous, mice exhibit retarded postnatal growth and symptoms of proprioceptive deficits, including clasping of the limbs when tail suspended and a hunched posture (Z. C. Baquet, unpublished observations). All animal procedures used to generate the *NT-3*^*lox-lacZ*^ strain were conducted in accord with US Public Health Service guidelines and with the approval of the University of Colorado Institutional Animal Care and Use Committee.

#### Characterization of SM22α^cre^-mediated recombination in NT-3^+/lox-lacZ^ reporter mice.

To map the spatial and temporal pattern of Cre-mediated recombination activity produced by the *SM22α*^*cre*^ transgene on the *NT-3*^*+/lox*^ allele, embryos were obtained from timed matings of *SM22α*^*cre/+*^;*NT-3*^*neo/+*^ × *NT-3*^*+/lox-lacZ*^ reporter mice, the same mating strategy used to generate *SM22α-NT-3*^*KO*^ mice. Also, ∼200-μm-thick brain slices from adult offspring from matings of *SM22α*^*cre/+*^ × *NT-3*^*+/lox-lacZ*^ reporter mice were obtained. Cre-mediated excision of floxed *NT-3* coding sequences in *NT-3*^*+/lox-lacZ*^ mice results in expression of the *lacZ* reporter gene under control of the *NT-3* promoter, similar to *BDNF*^+/*lox*^ mice (43). Thus β-galactosidase detected in tissues derived from these mice using X-gal staining identifies cells that transcribe the *NT-3* gene, but which have also lost the *NT-3* coding sequences. Thus analysis of β-galactosidase expression provides insights into the intersection of Cre recombinase transgene and *NT-3* gene expression in the conditional KO mice, implicating the origin of abnormal phenotypes due to the tissue-specific loss of NT-3 function. Noon of the day a copulatory plug was observed was designated E0.5 (mated females were checked for plugs at 0730). Embryos were harvested from E15–17, corresponding roughly to the middle of the period of NT-3 expression in GI smooth muscle (33, 36) and stained with X-gal. At each age, four to six bi- and tri-transgenic embryos from the *NT-3*^*+/lox-lacZ*^ matings were examined, and three control and three bi-transgenic adult brains were studied. The majority of embryos examined at a given age were derived from different mating pairs.

### Histochemical Staining of β-Galactosidase With X-gal

Embryos obtained from pregnant mice were prepared for X-gal staining and immediately fixed. Embryos were fixed on ice for 30 min with 1% paraformaldehyde (PF), 0.02% glutaraldehyde, 0.5 mM EGTA, and 2 mM magnesium chloride in 0.1 M sodium phosphate buffer, pH 7.4. Adult mice were given an overdose of methohexital sodium (120 mg/kg; Monarch Pharmaceuticals, Bristol, TN) and perfused with saline for 5–10 min until the liver cleared and then with ice-cold 4% PF for 30 min. The brain was immediately removed and postfixed for 1 h, and transverse sections ∼200 μm thick encompassing the entire brain and initial portion of the spinal cord were prepared. Embryos and brain slices were washed in three changes of buffer (2 mM magnesium chloride, 0.2% NP-40, and 0.1% sodium deoxycholate in 0.1 M sodium phosphate buffer, pH 7.4) on ice for 1 h total and stained overnight in the dark at 30°C in X-gal solution (5 mM potassium ferricyanide, 5 mM potassium ferrocyanide, and 0.1% X-gal in wash buffer; X-gal was dissolved at 2 mg/ml in dimethylformamide). Then embryos were postfixed for 48 h in 4% PF at 4°C, washed with PBS, and transferred to 10% buffered formalin at 4°C for a minimum of 5 days. Embryos were then embedded in paraffin, sectioned at a thickness of 8 μm, and air-dried on gelatin-coated slides. Alternate ribbons of sections were counterstained with 0.1% neutral red, and all sections were dehydrated in a series of graded alcohols (70%, 95%, 2 × 100%; 2 min each), cleared in xylene (3 × 2 min), and coverslipped with Cytoseal (Richard Alan Scientific, Kalamazoo, MI). Adult brain slices were stored in 4% PF at 4°C.

### Assessment of SM22α-NT-3^KO^ Effect on Vagal Signaling to the Brain Stem: Feeding-Induced c-Fos Activation in the DVC

#### Consumption of a larger-than-normal meal.

A protocol modified from Rinaman et al. (73) was used to reliably induce voluntary consumption of a larger-than-normal meal to activate vagal GI afferents and their target neurons in the DVC. *SM22α-NT-3*^*KO*^ (*n* = 18) and control (*SM22α*^*+/+*^;*NT-3*^*+/lox*^; *n* = 18) mice were each divided into two groups. For 5 consecutive days, mice were food deprived overnight, exposed to a palatable diet for 1 h each morning (Ensure Vanilla, 1.48 kcal/ml), followed by measurement of Ensure intake and body weight, and then fed chow for 3 h each afternoon. On the 5th day, at the time Ensure was normally offered, one group was given Ensure (*SM22α-NT-3*^*KO*^ fed, *n* = 8; control fed, *n* = 10), and the other was given no food (*SM22α-NT-3*^*KO*^ fasted, *n* = 10; control fasted, *n* = 8). One hour after food was offered, the weight of the food consumed was measured. Mutant and control mice of similar ages were run together to control for any inadvertent differences in laboratory environment or procedures that might have affected feeding behavior.

#### c-Fos response to consumption of a larger-than-normal meal.

Thirty minutes after the last 1-h presentation of Ensure (or no food), mice were perfused with fixative as described for adult mice used for X-gal staining of brain slices. Brains were removed and stored overnight at 4°C in the same fixative and switched to 25% sucrose in 0.1 M sodium phosphate-buffered saline, pH 7.4 (PBS) at 4°C for 48 h, and then 30-μm frozen cross sections were cut through the longitudinal extent of the DVC, and c-Fos was detected by immunohistochemistry. Immunohistochemistry procedures were performed at room temperature unless indicated. Every fifth section collected was stained with neutral red to aid identification of the borders of the nuclei that comprise the DVC, the nucleus of the solitary tract (NTS), area postrema (AP), and dorsal motor nucleus of the vagus (DMV) in adjacent c-Fos-stained sections. The remaining brain sections were washed in PBS, incubated 1 h in 0.3% H_2_O_2_, washed in PBS, incubated in blocking solution for 30 min (1.5% normal goat serum, 0.5% Triton X-100, 2% BSA), and then incubated 40–43 h in primary antibody (1:10,000; rabbit anti-c-Fos polyclonal no. PC38, Calbiochem, EMD Chemicals, Gibbstown, NJ) at 4°C. These, as well as the secondary antibody employed in the present experiments, were diluted with 1% BSA, 2% normal goat serum, and 0.3% Triton X-100. After washing with PBS, sections were incubated in secondary antibody for 45 min [biotin-conjugated goat anti-rabbit IgG (H+L); BA-1000; Vector Laboratories, Burlingame, CA], and then washed again with PBS, incubated in ABC reagent for 45 min (Vectastain Elite ABC kit PK-6100; Vector Laboratories), and exposed to a color reaction, involving 0.06% diaminobenzidine tetrahydrochloride, 0.075% H_2_O_2_, and 0.06% NiCl in Tris-buffered saline pH 7.6. Sections were washed in PBS, dehydrated, and cleared in 10-min changes of 70, 95, and 100% EtOH, and 45 min xylene, and coverslipped with Cytoseal. Brain sections of mutant and control mice were stained for c-Fos in parallel to control for any inadvertent variations in this procedure.

#### Quantification of c-Fos-like immunoreactivity.

Sections were mounted in the order collected from the caudal to rostral DVC. All sections with an intact DVC between the most caudal AP level and the obex (defined as the rostral-most section of the AP containing at least some AP tissue that extended completely across the caudal IVth ventricle) were used for quantification of c-Fos-like immunoreactivity (LIR). This resulted in 5–18 sections used per mouse, and the average number of sections used did not differ between fed and nonfed control and fed and nonfed mutant groups (not shown). AP, NTS, and DMV borders used to assign c-Fos-LIR neuronal nuclei to one of these brain structures were determined by comparison of each c-Fos-stained section with an adjacent neutral red-stained section from the same brain. Sections compared were separated by a distance of zero to two sections' thickness (0–60 μm) in the rostral or caudal direction. All stained elements approximating the size and shape of a cell nucleus were counted and recorded separately for each section, unless staining was too faint to clearly distinguish from background staining. To normalize these counts, for each mouse the average number of cells with a c-Fos-LIR-labeled nucleus per section was calculated and used for statistical analysis.

### Meal Pattern and Microstructure Analyses

*SM22α-NT-3*^*KO*^ (*n* = 11) and *SM22α*^*cre/+*^;*NT-3*^*+/lox*^ (*n* = 8) targeted KO mice and controls (*n* = 8) 3–4 mo of age were housed individually in plastic cages equipped with computerized pellet dispensers (Coulbourn Instruments, Allentown, PA). One *SM22α-NT-3*^*KO*^, one *SM22α*^*cre/+*^;*NT-3*^*+/lox*^, and one control mouse hoarded pellets during data collection, and, therefore, their data were not included in analyses, reducing the group sizes for *SM22α-NT-3*^*KO*^ (*n* = 10), *SM22α*^*cre/+*^;*NT-3*^*+/lox*^ (*n* = 7), and control (*n* = 7) mice.

#### Diet.

A balanced diet was employed (20 mg dustless precision pellets, BioServ, Frenchtown, NJ). The caloric distribution of this diet is 22% protein, 66% carbohydrate, and 12% fat, with a caloric density of 3.623 kcal/g. This is comparable to the maintenance diet in which the distribution is 28% protein, 60% carbohydrate, and 12% fat, with a caloric density of 3.04 kcal/g.

#### Experimental protocol and apparatus.

The balanced precision pellet diet was delivered using automated pellet dispensers and Graphic State software (version 2.0; Coulbourn Instruments), as described previously (35). Mice were adapted to the test room and test cages for 1 wk before testing. During that week, animals received three limited preexposures to the test diet to prevent neophobia at the start of testing, each consisting of 10 of the Bio-Serv precision pellets. Intake patterns were monitored 18 h each day. Animals were fasted the remaining 6 h, during which time cage maintenance was performed, and mice were weighed. Each daily session began at the start of the dark phase of the light cycle and extended 6 h into the light phase, and meal pattern data were collected for 22 consecutive days. This interval provided time for adaptation to the diets and apparatus, followed by at least 2 wk of stable intake patterns. Mice of each genotype were always tested in parallel to control for any inadvertent variations in the testing conditions.

#### Meal criteria.

Strict criteria were used to define a meal. Meal initiation was defined as a minimum of seven pellet removals with less than 20 min elapsing between responses. Once a meal was initiated, meal termination was defined as the onset of a 20-min interval with no intake. The criteria for meal onset (time interval between pellet removals and number of pellet removals) were determined by systematically varying them and examining the effect on meal number (35). These data were used to identify the range of criteria that exhibited the greatest stability in estimates of meal numbers, and the specific set of criteria chosen was drawn from the middle of this range. These criteria were applied to the raw data using the Graphic State software to identify the times of onset and termination of each meal, which were used to calculate several meal parameters. These were considered to be good estimates based on the observation that mice consumed all or almost all of each pellet, as evidenced by the minute amount of spillage present on cage floors.

#### Meal microstructure.

The first meal of each daily test session (defined as spontaneous food intake during the first 30 min after mice gained access to the food at the start of the session) was subjected to microstructural analysis to characterize changes in food intake rate over the course of this meal (18). Initial intake rate and changes in this rate across the 30-min feeding session were estimated by determining the amount of food consumed during each minute of the 30-min meal. The rationale for this approach has been discussed in detail previously (35).

### Microscopy and Imaging

X-gal- and c-Fos-stained tissue were examined with standard bright-field or differential interference contrast illumination (Leica DM5000 microscope). Photomicrographs were acquired directly with a video camera (Spot RT Slider; Diagnostic Instruments, Sterling Heights, MI).

### Statistical Analysis and Graphical Display of Data

In all experiments and genotype groups, male and female mice were included, but most groups studied included greater numbers of males. Nevertheless, there were no significant sex differences within any genotype in body weight, or any meal pattern or microstructure measure (not shown). Therefore, data from males and females of the same genotype were combined for all analyses. Group data with males and females combined were normally distributed, and groups that were compared exhibited similar variance (not shown).

The body weights of mice measured at the start of training for the meal-induced c-Fos experiment and food intake on the test day were compared between controls and *SM22α-NT-3*^*KO*^ mice using *t*-tests. Baseline c-Fos activation (c-Fos-LIR cell numbers/section in nonfed mice) and meal-induced c-Fos activation (c-Fos-LIR cell numbers/section in fed mice) in the DVC of control and *SM22α-NT-3*^*KO*^ mice in each brain region were also compared using *t*-tests. For a small number of these comparisons, group variances were not equal. In these instances, Welch's correction was employed. For meal pattern data, including food intake and each meal pattern parameter, planned pairwise comparisons between heterozygous KOs (*SM22α*^*cre/+*^;*NT-3*^*+/lox*^) and controls, or between homozygous KOs (*SM22α-NT-3*^*KO*^) and controls were tested using one-way ANOVA with repeated measures over *days 7–22* and genotype as the independent variable. Regarding first meal microstructural parameters, differences in food intake at *minute 1* were examined using one-way ANOVA with genotype as the independent variable, and the Tukey post hoc test was employed. Comparisons of decay of eating rate were tested using one-way ANOVA with repeated measures over *minutes 2–4* and *5–30* with genotype as the independent variable. Values reported are means ± SE. For all statistical tests, *P* < 0.05 was required for statistical significance. Statistica (version 5.0, StatSoft, Tulsa, OK) was employed for all statistical comparisons that used ANOVA. Graphpad was utilized for comparisons that used *t*-tests and to construct all graphs (Graphpad Prism version 4.0, Graphpad Software). Photoshop CS software (version 8.0 Adobe Systems, Mountain View, CA) was used to apply scale bars and text to digital photomicrographs, adjust their brightness and contrast, and organize final figure layouts.

## RESULTS

### Extent and Specificity of Smooth Muscle-Specific NT-3 KO: Assessment of SM22α^cre^-Mediated Recombination

The structure of the different *NT-3* alleles used here allows both the analysis of *NT-3* gene expression (using *NT-3*^*lacZ*^), and identification of the populations that lose NT-3 function due to Cre-mediated recombination (using *NT-3*^*lox-lacZ*^). *NT-3* expression in the GI tract is illustrated by *β-galactosidase* staining in E15–17 embryos derived from *NT-3*^*lacZ*^ mice (Fig. 1, *B*–*E*). NT-3 is expressed in mid-to-late gestation embryos in smooth muscle of the outer wall of the stomach, intestines, cecum, as well as in smooth muscle of blood vessels that supply these organs (33, 36). In the GI organs, this expression occurs throughout the circular (inner) smooth muscle layer, whereas it is more restricted in the longitudinal (outer) smooth muscle layer, occurring only in some regions of the stomach and large intestine. At mid-late gestation, NT-3 was also expressed in the lamina propria of the stomach wall and the epithelium of the esophagus.

The extent and specificity of *SM22α*^*cre*^-mediated recombination activity on the *NT-3*^*+/lox*^ allele, and in *NT-3* expressing cell populations, were assessed in *SM22α*^*cre/+*^;*NT-3*^*+/lox-lacZ*^ and *SM22α-NT-3*^*KO*^ embryos derived from matings of *SM22α*^*cre/+*^ × *NT-3*^*neo/lox-lacZ*^ mice (Fig. 1, *B*′–*E*″). This analysis identified tissues from which *NT-3* function was lost as a result of this recombination in *SM22α*^*cre/+*^;*NT-3*^*+/lox-lacZ*^ and *SM22α-NT-3*^*KO*^ mice. In *SM22α*^*cre/+*^;*NT-3*^*+/lox-lacZ*^ and *SM22α-NT-3*^*KO*^ embryos, X-gal staining was examined from E15 to E17. These analyses documented that recombination occurred in nearly all GI tissues that normally produce NT-3, including the smooth muscle of the stomach, small intestine, cecum, large intestine, and GI blood vessels. Furthermore, within all of these tissues, except for the stomach (see below), Cre-mediated recombination appeared to encompass the majority of cells normally expressing NT-3. Much of the circular layer of smooth muscle and the longitudinal smooth muscle layer in some regions of stomach and large intestine (e.g., arrow in Fig. 1*B*″), which normally express NT-3, underwent recombination. In contrast, the esophageal epithelium and lamina propria of the stomach did not exhibit recombination. This reduced spatial extent of β-galactosidase in the GI tract of *SM22α*^*cre/+*^;*NT-3*^*+/lox-lacZ*^ and *SM22α-NT-3*^*KO*^ embryos compared with *SM22α*^*cre/+*^;*Rosa26* embryos (34) and, compared with the extent of NT-3 expression in *NT3*^*lacZ*^ embryos (33, 36), was consistent with detection of β-galactosidase being restricted not only by the *SM22α*^*cre*^ promoter (as in *SM22α*^*cre/+*^;*Rosa26* mice), but also by the *NT-3* promoter. Additionally, although NT-3 is normally expressed in one or both smooth muscle layers throughout almost the entire stomach wall, only a small proportion of the smooth muscle layers of the stomach wall exhibited X-gal staining indicative of Cre-mediated recombination. This reduced spatial extent of Cre-mediated recombination in the stomach smooth muscle was consistent with our laboratory's previous observation that the *SM22α*^*cre*^ transgene only produced recombination in about one-fourth to one-third of the stomach wall, as assessed in *SM22α*^*cre/+*^;*Rosa26* embryos (34). Direct comparisons of the patterns of NT-3 expression and Cre-mediated recombination activity on the *NT-3*^*lox-lacZ*^ allele are illustrated for each organ system in Fig. 1. Specifically, for the stomach, compare NT-3 expression illustrated in Fig. 1*B* with Cre-mediated recombination shown in Fig. 1, *B*′ and *B*″; for the intestines compare NT-3 expression in Fig. 1*C* with Cre-mediated recombination in Fig. 1, *C*′ and *C*″; for the cecum compare NT-3 expression in Fig. 1*D* with Cre-mediated recombination in Fig. 1, *D*′ and *D*″; for blood vessels traversing intestinal mesentery compare *NT-3* expression in Fig. 1*E* with Cre-mediated recombination in Fig. 1, *E*′ and *E*″.

**Fig. 2.**
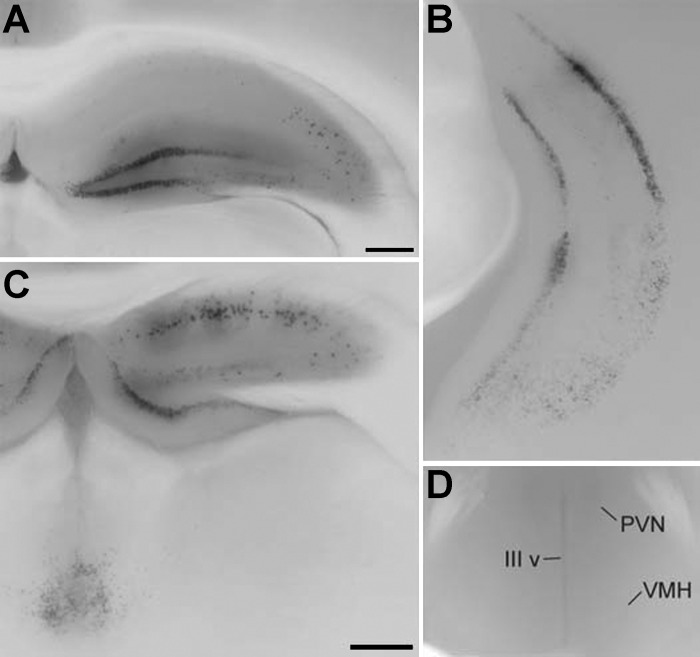
Cre-mediated recombination in *SM22α*^*cre/+*^;*NT-3*^*+/lox-lacZ*^ mice occurred within a small number of adult brain regions, including the hippocampus (*A*–*C*) and intermediodorsal thalamus (*C*). Photomicrographs of coronal brain slices taken from adult *SM22α*^*cre/+*^;*NT-3*^*+/lox-lacZ*^ mice that were stained with X-gal. *A*: cross section through the dorsal hippocampus. *B*: cross section through the ventral hippocampus. *C*: section that includes a different anterior-posterior level of the dorsal hippocampus (*top* portion of image) compared with *A*, and the intermediodorsal thalamus (*bottom left* portion of image). *D*: cross section through the hypothalamus. There was no evidence of recombination in the hypothalamus as shown here, or in the brain stem, including the dorsal vagal complex (DVC; not shown). PVN, paraventricular nucleus; VMH, ventromedial hypothalamus; IIIv, third ventricle. Scale bars in *A* and *C* = 400 μm, and the bar in *C* also applies to *B* and *D*.

At the embryonic ages studied, there was no evidence of recombination in the brain, consistent with the similar finding of lack of evidence of recombination in the brains of *SM22α*^*cre/+*^;*Rosa26* and *SM22α*^*cre/+*^;*BDNF*^*neo/lox*^ embryos (Ref. 34 and not shown). Regarding the adult brain, the *SM22α*^*cre/+*^ Cre driver strain we employed has been described as producing recombination only in smooth muscle (57). We recently found evidence, however, that it also produces recombination in the adult brain (34). Therefore, we examined the adult brain of *SM22α*^*cre/+*^;*NT-3*^*+/lox-lacZ*^ mice, and, in fact, there was staining suggestive of Cre-mediated recombination, but it was limited to three highly circumscribed regions. These included the hippocampus (Fig. 2, *A*–*C*), a small group of cells in the region of the intermediodorsal thalamic nucleus (Fig. 2*C*), and another group in the region of the caudomedial entorhinal cortex (not shown). There was no evidence of recombination in the hypothalamus (Fig. 2*D*) or brain stem, including the DVC (not shown).

Overall, these results suggested during development the *SM22α*^*cre*^ transgene targeted the KO of *NT-3* to smooth muscle of the GI wall and vasculature with high selectivity. They also implied that this KO occurred sufficiently early in development to largely eliminate NT-3 expression at ages when vagal axons are in the process of growing into the upper GI tract (begins on E12 and continues through early postnatal ages; Ref. 65) and mechanoreceptor terminals are forming (begins on E16 and continues through early postnatal ages; Ref. 65). These estimates are based largely on development of stomach innervation; development of intestinal innervation is likely to be slightly delayed relative to this. The present characterization of *SM22α*^*cre*^-mediated recombination of the *NT-3*^*+/lox*^ allele further suggests the *NT-3* KO in smooth muscle was largely complete in the intestines, whereas it appeared to be largely incomplete in the stomach. This could imply any effect of NT-3 loss from smooth muscle on development of vagal GI afferents or other GI innervation should have been most prominent for neural pathways that supply the intestines.

### Assessment of Vagal Afferents That Innervate the GI Tract in SM22α-NT-3^KO^ Mice: Activation of Nuclei Within the DVC by Meal-Related Stimuli

We hypothesized that reduced levels of NT-3 in GI smooth muscle would disrupt development of the vagal GI afferents. If this occurred, one consequence would be vagal signaling from the gut to the brain would be reduced. To examine signaling by vagal afferents that innervate the upper GI tract in *SM22α-NT-3*^*KO*^ mice, meal-induced activation of the *c-Fos* immediate early gene in the brain stem nuclei that receive direct input from vagal GI afferents was compared in *SM22α-NT-3*^*KO*^ and control mice. If negative feedback signaling by vagal GI afferents was reduced in *SM22α-NT-3*^*KO*^ mice, then meal-induced activation of their NTS neurons, and possibly of their AP or DMV neurons, should have been decreased. A larger-than-normal meal was employed to activate as large a proportion of as many types of vagal afferents that innervate the upper GI tract as possible to increase the probability of detecting altered signaling. Furthermore, large liquid meals have produced larger or more reliable increases in c-Fos activation in the NTS and AP compared with smaller meals (25, 73).

**Fig. 3.**
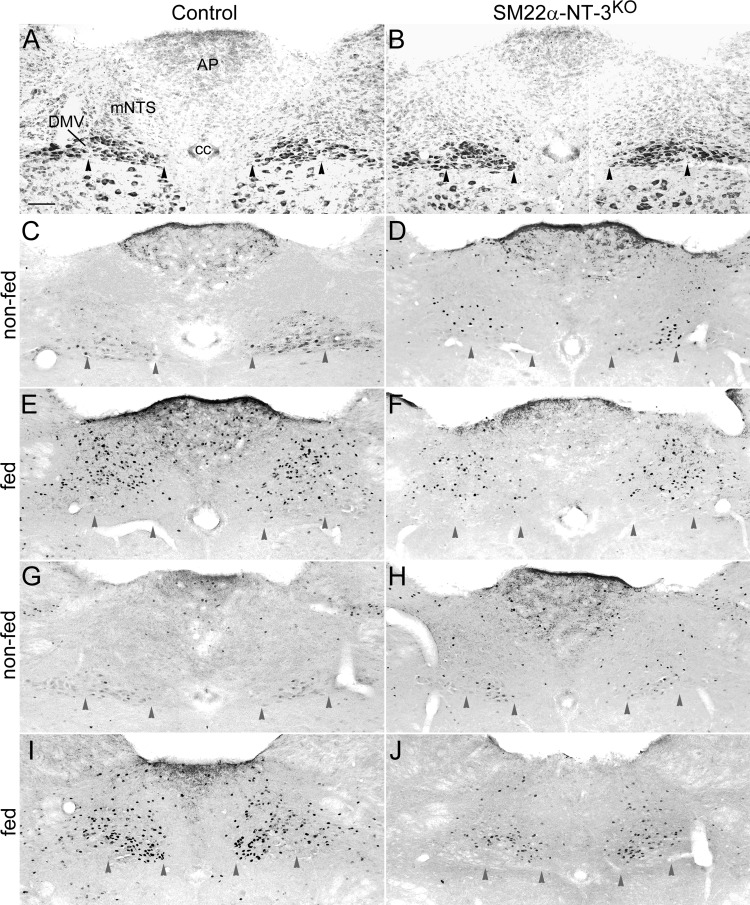
Meal-induced c-Fos activation was increased in the area postrema (AP) and nucleus of the solitary tract (NTS) of *SM22α-NT-3*^*KO*^ mice compared with controls. Cross sections are through the DVC of controls (*A*, *C*, *E*, *G*, *I*) and *SM22α-NT-3*^*KO*^ mice (*B*, *D*, *F*, *H*, *J*). *A* and *B*: sections taken from AP levels of the DVC sampled and stained with neutral red are illustrated. *C*–*J*: sections taken from anterior (*C*–*F*) and mid-caudal (*G*–*J*) AP levels of the DVC sampled and stained for c-Fos-like immunoreactivity (LIR) are shown. Arrowheads indicate the approximate medial and lateral borders of the gastric (medial) column of dorsal motor nucleus of the vagus (DMV) neurons, and they point to the approximate ventral border of the DMV. *C*, *D*, *G*, *H*: few cells showed c-Fos-LIR in the nonfed groups. *E*, *F*, *I*, *J*: increased numbers of c-Fos-LIR neuronal nuclei were observed in the medial subnucleus of the NTS (mNTS), DMV, and AP of the fed groups. *I* and *J*: in the DMV, this increase was concentrated in the gastric column of neurons at mid-caudal AP levels. Scale bar in *A* = 100 μm, applies to all images. cc, Central canal.

**Fig. 4.**
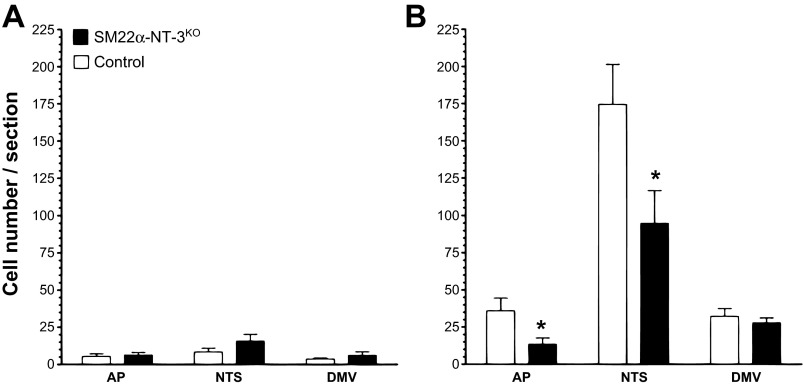
Quantification of c-Fos-LIR in the DVC of control and *SM22α-NT-3*^*KO*^ mice. *A*: the average number of c-Fos-LIR neuronal nuclei stained per section in nonfed control and *SM22α-NT-3*^*KO*^ groups in the AP, mNTS, and DMV is plotted. There were no group differences. *B*: the average number of c-Fos-LIR neuronal nuclei stained per section in fed control and *SM22α-NT-3*^*KO*^ groups in the AP, mNTS, and DMV is plotted. In the AP and NTS, c-Fos-LIR was reduced in *SM22α-NT-3*^*KO*^ mice compared with controls. *Statistically significant.

Examples of Nissl-stained sections of the DVC sampled are illustrated in a control and an *SM22α-NT-3*^*KO*^ mouse (Fig. 3, *A* and *B*, respectively). Qualitative examination of Nissl-stained brain sections suggested the structure of the AP, NTS, and DMV were similar in *SM22α-NT-3*^*KO*^ and control mice. Representative c-Fos responses are shown in control and *SM22α-NT-3*^*KO*^ mice from the nonfed groups (Fig. 3, *C* and *G*, and *D* and *H*, respectively) and the fed groups (Fig. 3, *E* and *I*, and *F* and *J*, respectively). Baseline c-Fos activation (number of cells with c-Fos-LIR nuclei in nonfed groups) was similar in *SM22α-NT-3*^*KO*^ and control mice for all three brain regions studied: the AP, NTS, and DMV (*P* = 0.34, 0.10, and 0.16, respectively; Fig. 4*A*). Consumption of a large meal increased the number of c-Fos positive cells in controls and mutants, respectively, in the AP (681%, *P* < 0.01; 213%, *P* = 0.06), NTS (2,095%, *P* < 0.01; 606%, *P* < 0.01), and DMV (2,065%, *P* < 0.01; 458%, *P* < 0.01), although this increase failed to reach significance for *SM22α-NT-3*^*KO*^ mice in the AP (cf. Fig. 4, *A* and *B*). Consistent with the hypothesis that vagal afferent signaling was reduced in *SM22α-NT-3*^*KO*^ mice, they exhibited a reduction in meal-induced c-Fos activation in the NTS and AP compared with controls (46% decrease, *P* < 0.05; 63% decrease, *P* < 0.05, respectively; Fig. 4*B*), whereas there was no change in response in the DMV (13% decrease; *P* = 0.15; Fig. 4*B*). There were no differences in body weight at the start of the c-Fos experiment (controls, 24.04 ± 1.51 g; *SM22α-NT-3*^*KO*^ mice, 24.43 ± 0.1 g) or in test meal size (controls, 3.99 ± 0.24 g; *SM22α-NT-3*^*KO*^ mice, 4.1 ± 0.27 g) between controls and *SM22α-NT-3*^*KO*^ mice. This suggested it is unlikely that these factors contributed to the group differences in c-Fos activation within the NTS and AP. Since the meal pattern analysis described below detected a decrease in the rate of intake in the *SM22α-NT-3*^*KO*^ mice, however, it is possible this contributed to their reduction in feeding-induced c-Fos activation (see the discussion for elaboration).

### Effect of SM22α-NT-3^KO^ on Body Weight and Feeding Behavior

Here we examined whether the reduction of vagal signaling of meal-related stimuli to the NTS and AP in *SM22α-NT-3*^*KO*^ mice reported above was sufficient in magnitude to be detected utilizing sensitive measures of feeding behavior. One of the major functions of signaling to the brain by vagal afferents that innervate the GI tract is typically considered to be negative feedback signaling, which contributes to meal termination, or satiation. Therefore, we hypothesized that, if vagal afferent activation was sufficiently curtailed in *SM22α-NT-3*^*KO*^ mice, meal parameters would be altered in a manner consistent with reduced satiation.

#### Daily body weight and food intake.

**Fig. 5.**
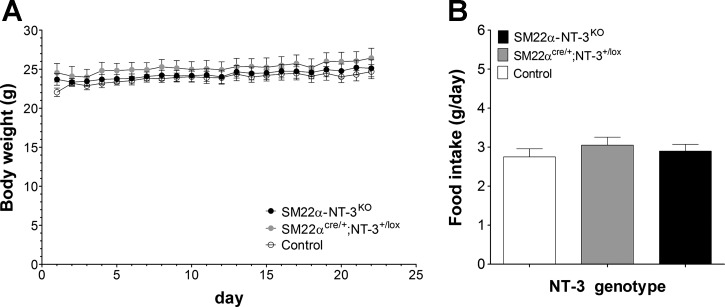
*SM22α-NT-3*^*KO*^ and *SM22α*^*cre/+*^;*NT-3*^*+/lox*^ mice exhibited similar mean body weight and food intake as controls at 3–4 mo of age. *A*: growth curves plotted for these three groups over the 22 days of meal pattern data collection. *B*: average food intake for each group over *days 7–22* of meal pattern data collection is plotted.

Before testing this hypothesis, we examined whether manipulation of GI NT-3 altered long-term regulation of food intake and body weight, effects that could confound interpretation of meal patterns. During meal pattern data collection (12–16 wk of age), the average daily body weights and food intakes of *SM22α-NT-3*^*KO*^, *SM22α*^*cre/+*^;*NT-3*^*+/lox*^, and control mice were similar: the average body weight of each of the three groups throughout meal pattern testing was stable at ∼23–25 g (*P* = 0.32; Fig. 5*A*). The average amount of food consumed daily by animals in each group was ∼2.8–3.0 g of the BioServ diet (*P* = 0.6; Fig. 5*B*).

#### Meal patterns.

In a test of the hypothesis that vagal afferent activation is reduced in mice with smooth muscle-specific KO of *NT-3*, we performed an analysis of several meal-related parameters. Eating patterns of *SM22α-NT-3*^*KO*^, *SM22α*^*cre/+*^;*NT-3*^*+/lox*^, and control mice stabilized by *day 7*; therefore, data obtained from *days 7–22* were analyzed. Ensuring the food intake patterns had stabilized should have minimized the influence of any potential differences in learning ability between mutants and controls that could possibly have resulted due to reduced NT-3 levels in the hippocampus. The averaged values for each meal parameter for each group that are not graphed are listed in Table 1.

**Table 1.** Meal pattern parameters that did not exhibit any significant differences between groups

	Control	*SM22α*^*cre/+*^;*NT-3*^*+/lox*^	*SM22α-NT-3*^*KO*^
Meal size, g	0.329 ± 0.019	0.308 ± 0.019	0.334 ± 0.016
Meal frequency, mean no./day	9.89 ± 0.53	10.34 ± 0.55	9.39 ± 0.76
Average IMI, min	58.29 ± 1.96	62.91 ± 2.95	63.86 ± 2.99
Total IMI, min	594.8 ± 56.54	641.57 ± 46.05	590.63 ± 47.13
Satiety ratio, min/g	216.14 ± 25.14	212.22 ± 22.78	207.36 ± 21.78

	Control vs. *SM22α*^*cre/+*^;*NT-3*^*+/lox*^	Control vs. *SM22α-NT-3*^*KO*^	*SM22α*^*cre/+*^;*NT-3*^*+/lox*^ vs. *SM22α-NT-3*^*KO*^
Meal size	0.477	0.860	0.206
Meal frequency	0.183	0.721	0.373
Average IMI	0.353	0.542	0.829
Total IMI	0.533	0.956	0.468
Satiety ratio	0.853	0.669	0.800

Values are group means ± SE (*top*) and *P* values for pairwise comparisons (*bottom*). Values are based on the average of these daily values over the last 16 days of behavioral testing (*days 7–22*). IMI, intermeal interval; NT-3, neurotrophin-3; KO, knockout.

**Fig. 6.**
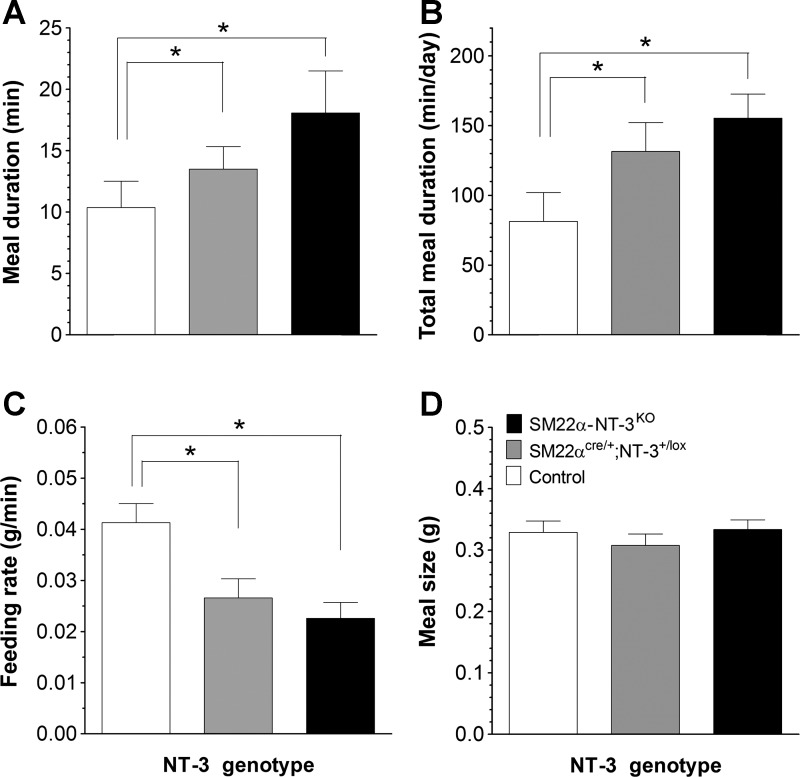
Altered meal patterns of *SM22α-NT-3*^*KO*^ mice suggested they had a deficit in satiation, or meal termination signaling. *A*: *SM22α*^*cre/+*^;*NT-3*^*+/lox*^ and *SM22α-NT-3*^*KO*^ mice exhibited 30 and 70% increases in average meal duration compared with the control group, respectively. *B*: *SM22α*^*cre/+*^;*NT-3*^*+/lox*^ and *SM22α-NT-3*^*KO*^ mice showed 61 and 94% increases in the average total daily meal duration compared with controls, respectively. *C*: *SM22α*^*cre/+*^;*NT-3*^*+/lox*^ and *SM22α-NT-3*^*KO*^ mice exhibited 36 and 45% decreases in average feeding rate compared with controls, respectively. *D*: average meal size of *SM22α-NT-3*^*KO*^ and *SM22α*^*cre/+*^;*NT-3*^*+/lox*^ mice did not differ from controls because their decreases in eating rate (*C*) largely compensated for their increases in meal duration (*A* and *B*). *Significant difference.

*SM22α-NT-3*^*KO*^ and *SM22α*^*cre/+*^;*NT-3*^*+/lox*^ mice demonstrated highly selective effects on meal parameters: both mutant groups exhibited increases in meal duration relative to controls. In particular, the average meal durations of *SM22α*^*cre/+*^;*NT-3*^*+/lox*^ and *SM22α-NT-3*^*KO*^ mice were increased by 30 and 70%, respectively, compared with control mice (both *P* < 0.05; Fig. 6*A*). Furthermore, over the course of each day, the average total meal duration of *SM22α*^*cre/+*^;*NT-3*^*+/lox*^ and *SM22α-NT-3*^*KO*^ mice increased by 61 and 94%, respectively, compared with controls (both *P* < 0.05; Fig. 6*B*). These increases in meal duration, however, did not lead to an increase in average meal size because the targeted KO mice compensated by decreasing their rate of food intake. Specifically, *SM22α-NT-3*^*KO*^ mice demonstrated a 45% decrease in average consumption rate compared with controls, and *SM22α*^*cre/+*^;*NT-3*^*+/lox*^ mice a 36% reduction (both *P* < 0.05; Fig. 6*C*). Consequently, average meal size was similar for all three groups, equal to slightly more than one-third of a gram per meal (Table 1; Fig. 6*D*). While these effects of local NT-3 deficiency on average and total meal duration and average eating rate all showed trends toward a dependence on gene-dosage, the group differences between *SM22α-NT-3*^*KO*^ and *SM22α*^*cre/+*^;*NT-3*^*+/lox*^ mice were not significant (*P* = 0.31, *P* = 0.45, and *P* = 0.32, respectively). This suggests that the effect of the global loss of one *NT-3* allele on meal duration and eating rate was not additive with the effect of loss of the other *NT-3* allele from a large percentage of GI smooth muscle cells. Average daily meal number was also similar for *SM22α-NT-3*^*KO*^, *SM22α*^*cre/+*^;*NT-3*^*+/lox*^, and control mice, as they all consumed an average of 9 or 10 meals a day (Table 1). Furthermore, there were no differences in average intermeal interval (IMI; Table 1) or satiety ratio (ratio of meal size to the subsequent IMI; Table 1) between groups. These similarities between groups in meal number, IMI, and satiety ratio suggested satiety (the effect of food consumed during a meal on the delay until initiation of the next meal) and other factors that can affect meal initiation were not altered by the targeted *NT-3* mutations.

#### Meal microstructure.

**Fig. 7.**
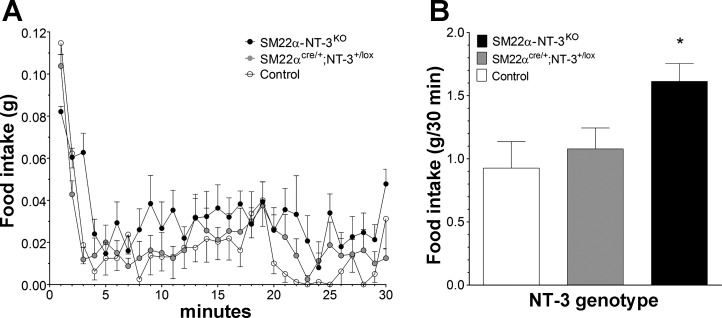
Microstructural analysis of the first 30 min of food intake each day was consistent with a satiation deficit in *SM22α-NT-3*^*KO*^ mice. *A*: during the first minute of the first daily meal (first 30 min of food access), which was used as an estimate of the initial intake rate, *SM22α-NT-3*^*KO*^ mice demonstrated a significant reduction in food intake compared with the control group, suggesting they had a lower initial intake rate. This could imply that they had reduced oropharyngeal-positive feedback, which drives food intake. During the late component of decay of eating rate (*minutes 5–30*), *SM22α-NT-3*^*KO*^ mice exhibited a nonsignificant trend toward a reduced rate of decay of food intake compared with the control group. Additionally, *SM22α-NT-3*^*KO*^ and *SM22α*^*cre/+*^;*NT-3*^*+/lox*^ mice ate continuously throughout the 30 min, whereas controls terminated feeding as early as *minute 22*, consistent with reduced satiation signaling in the mutant groups. *B*: for *SM22α-NT-3*^*KO*^ mice, this continuous eating combined with their trend toward a reduction in the late component of the decay of eating rate resulted in an increase in the size of the first meal compared with controls. *Significant difference.

To gain additional evidence in support of the interpretation that disruption of vagal negative feedback signaling from gut to brain, or satiation signaling, contributed to the increased meal duration of *SM22α-NT-3*^*KO*^ mice, microstructure of the first 30 min of food intake on *days 7–22* of meal pattern data collection was examined. Three microstructural parameters are often used to characterize changes in the rate of eating over the course of a meal (18, 35): *1*) the initial consumption rate, which is mainly influenced by oropharyngeal stimulation; *2*) the early component of decay of eating rate, which is influenced by both oropharyngeal positive feedback and vagal GI negative-feedback signaling; and *3*) the late component of decay of eating rate, which is mainly influenced by vagal GI negative feedback. All three groups of mice exhibited high rates of food intake during the first minute of access to food (estimate of initial intake rate), but that of the *SM22α-NT-3*^*KO*^ mice was lower than it was for controls (*P* < 0.05). This could imply the food was less palatable for *SM22α-NT-3*^*KO*^ mice, generating less oropharyngeal-positive feedback than in controls. After the first minute of food access, eating rate rapidly declined and then leveled off by *minutes 4–5*. Thus *minutes 2–4* were used to estimate the early component of decay of eating rate and the remainder of the 30 min (*minutes 5–30*) to estimate the late component. For *minutes 5–30* of the first meal, *SM22α-NT-3*^*KO*^ and *SM22α*^*cre/+*^;*NT-3*^*+/lox*^ mice ate continuously, albeit at a slow rate, whereas controls, which also ate at a slow rate, stopped feeding by *minute 22*. This pattern of feeding in the *SM22α-NT-3*^*KO*^ and *SM22α*^*cre/+*^;*NT-3*^*+/lox*^ mice was unusual. Control mice in this study and in a previous one that utilized the same scheduled meal protocol as employed here stopped eating within the first 15–22 min after initial access to food (34). Thus, although there were no significant differences in eating rate between either of the mutant groups and controls during the early (*minutes 2–4*) or late (*minutes 5–30*) components of decay of eating rate (Table 2), the continuous eating throughout the first meal exhibited by *SM22α-NT-3*^*KO*^ and *SM22α*^*cre/+*^;*NT-3*^*+/lox*^ mice was consistent with a contribution of decreased satiation signaling to their increase in average meal duration. Also consistent with this interpretation, the continuous eating of *SM22α-NT-3*^*KO*^ mice throughout the first 30 min of food access each day combined with their trend toward reduced decay of eating rate from *minutes 5–30* resulted in a 74% increase in their first meal size compared with controls (*P* < 0.05: Fig. 7, *A* and *B*). Finally, the lower rate of eating exhibited by *SM22α-NT-3*^*KO*^ mice compared with controls during *minute 1* of the first meal, which estimates the initial intake rate, suggests that the increased oropharyngeal-positive feedback signaling that drives food intake was reduced in *SM22α-NT-3*^*KO*^ mice. This indicates that increased oropharyngeal positive feedback did not contribute to their increase in meal size. Since vagal negative feedback is the only other major signal contributing to the control of meal size, the lack of a contribution by oropharyngeal positive feedback is consistent with reduced negative feedback being the main cause of increased meal size in *SM22α-NT-3*^*KO*^ mice.

**Table 2.** Microstructure data for early and late components of decay of eating rate during the first 30 min of food access each day

Decay of Eating Rate	Control	*SM22α*^*cre/+*^;*NT-3*^*+/lox*^	*SM22α-NT-3*^*KO*^
*Means ± SE, g/min*			
Early component (*minutes 2–4*)	0.012 ± 0.004	0.006 ± 0.002	0.014 ± 0.004
Late component (*minutes 5–30*)	0.0025 ± 0.003	0.003 ± 0.0003	0.004 ± 0.0003
*P values*			
Control		0.068	0.321
*SM22α*^*cre/+*^;*NT-3*^*+/lox*^	0.373		0.040*
*SM22α-NT-3*^*KO*^	0.149	0.265	

Values are group means ± SE (*top*) and *P* values for pairwise comparisons (*bottom*). The tabled values are based on the average of these daily values over the last 16 days of behavioral testing (*days 7–22*).

* Statistically significant.

## DISCUSSION

In the present study, the cre-lox recombination method was used to reduce NT-3 expression in GI smooth muscle, a tissue densely innervated by vagal mechanoreceptors. Evaluation of *SM22α*^*cre*^-mediated recombination in NT-3-expressing populations indicated the *NT-3* gene was deleted with high efficiency from the smooth muscle of the wall of the intestines, cecum, a small portion of the stomach, and blood vessels that supply the gut. Meal-induced c-Fos activation was reduced in the NTS and AP, but not the DMV of *SM22α-NT-3*^*KO*^ mice compared with controls. This suggested vagal negative feedback signaling from the upper GI tract to the brain was decreased in *SM22α-NT-3*^*KO*^ mice and could imply vago-vagal reflexes were not significantly affected. Examination of meal pattern and microstructure detected effects on short-term feeding behavior that were consistent with reduced vagal negative feedback signaling to the brain, or decreased satiation signaling. In particular, the primary effect of reduced smooth muscle NT-3 expression was to increase meal duration. *SM22α-NT-3*^*KO*^ and *SM22α*^*cre/+*^;*NT-3*^*+/lox*^ mice had increases in average meal duration compared with controls, which accumulated over the course of each day to also result in increases in total daily meal duration. Despite the increase in meal duration of both *SM22α-NT-3*^*KO*^ and *SM22α*^*cre/+*^;*NT-3*^*+/lox*^ mice, neither group exhibited an increase in meal size because they compensated by decreasing their rates of food intake. Consistent with this compensation, daily food intake and body weight were similar in mutant and control mice. This suggested no long-term effects on feeding behavior appeared to be associated with decreased NT-3 levels in GI smooth muscle or with the associated reduction in meal-induced negative feedback signaling. To further examine whether the increase in meal duration was reflective of reduced vagal satiation signaling, microstructure of the first daily meal was characterized. Although trends in the decay of eating rate over the course of this first meal in mutants compared with controls were in a direction consistent with reduced negative feedback, they were not significant. *SM22α-NT-3*^*KO*^ mice, however, ate continuously for the entire 30 min of food access, whereas controls terminated feeding by *minute 22*. Moreover, the nonsignificant decrease in rate of decay of eating in *SM22α-NT-3*^*KO*^ mice compared with controls, and their continuous eating over the initial 30 min of food access, combined to produce a large increase in first meal size compared with controls. The reduction in the meal-induced c-Fos activation in the NTS and AP and the increases in average meal duration and first meal size of the *SM22α-NT-3*^*KO*^ mice taken together are consistent with reduced satiation signaling by vagal afferents.

The most parsimonious hypothesis to account for the aberrant feeding patterns in mice with reduced NT-3 expression in GI smooth muscle is that decreased target-derived NT-3 disrupted development of vagal GI afferents, which led to reduced satiation signaling. As described in the Introduction, a large proportion of vagal sensory neurons fail to survive in *NT-3* global KO mice (27, 29, 58, 84). Critically, the only vagal sensory neurons identified to date that are dependent on NT-3 for survival are esophageal vagal mechanoreceptors, and the loss of these accounts for only a small proportion of the missing vagal afferents: there were ∼130 esophageal IGLEs missing vs. over 2,800 vagal sensory neurons lost from heterozygous *NT-3* KO mice (71). Additionally, in other sensory systems, NT-3 produced by peripheral tissues influences the survival of various classes of mechanoreceptors. For example, cutaneous overexpression of NT-3 increases the number of DRG neurons and the innervation of touch domes and hair follicles (2). Even when NT-3 loss from the region of the sensory ganglion is essential for survival at early stages of development, NT-3 produced by peripheral tissues has been found essential for mechanoreceptor survival after target tissue innervation. For example, blocking the effects of NT-3 produced by muscle cells with NT-3-specific antiserum prevented the survival of sensory neurons with large diameters, including muscle spindle afferents (66). Moreover, although there are other possible effects of the smooth muscle-specific *NT-3* KO that could have mediated, or contributed to, the aberrant feeding patterns, evaluation of these possibilities described below suggests they are either unlikely, or they could not entirely account for the present findings. Therefore, this evaluation is consistent with the hypothesis outlined above.

Because *SM22α-NT-3*^*KO*^ mice exhibited a reduced eating rate during the meal pattern analysis, it is possible a decrease in feeding rate contributed to the group differences in meal-induced c-Fos activation. Several observations taken together, however, suggest this was not likely to have been a major factor. Although *SM22α-NT-3*^*KO*^ mice had a reduced eating rate during the meal pattern analysis, mild food deprivation (6 h during the light portion of the circadian cycle) eliminated the group differences in eating rate during the first daily meal (Fig. 7*A*). In contrast, the effect on meal duration was maintained (Fig. 7*A*). As the c-Fos test meal followed an even longer period of food deprivation than the first daily meal (overnight vs. 6 h) and a more palatable diet was employed (liquid Ensure vs. BioServ pellet balanced diet), it is probable again that rate of feeding of the *SM22α-NT-3*^*KO*^ mice would have been normalized. Consistent with this possibility, during the test meal we observed that all mice examined for c-Fos activation consumed the test diet in about the same timeframe, suggesting their average intake rates were similar. Nevertheless, since we did not quantify these feeding rates, we cannot rule out the possibility that group differences in this rate contributed to the lower-than-normal meal-induced c-Fos activation in the NTS and AP of *SM22α-NT-3*^*KO*^ mice. Importantly though, available evidence suggests that, even if a group difference in eating rate occurred, its effect on c-Fos activation would have been minor. For example, reducing the gastric infusion rate of a liquid diet by a factor of 10 caused a 55% decrease in c-Fos activation in the NTS (18). If this effect is linear, it could imply the reduction in eating rate in *SM22α*-*NT-3*^*KO*^ mice during meal pattern analysis, which was by a factor of 0.45, would translate to less than a 3% decrease in their c-Fos activation. This decrease is minor compared with the size of the decreases in meal-induced c-Fos activation in *SM22α-NT-3*^*KO*^ mice, which were 54% for the NTS and 67% for the AP.

### Alternative Pathways/Mechanisms Contributing to Altered Meal Patterns of SM22α-NT-3^KO^ Mice

#### Altered function of mature vagal afferents that supply the GI tract.

One alternative mechanism that could have led to reduced vagal satiation signaling in *SM22α-NT-3*^*KO*^ mice is a direct effect of reduced NT-3 levels in adult GI smooth muscle on maintenance or function of vagal afferents. In fact, NT-3 has been shown to have such effects in other peripheral sensory systems. These include effects on mechanoreceptor maintenance for a class of skin afferents (3) and on neurotransmission by muscle spindle afferents in neonates (68), as well as on inner hair cells and thermally sensitive DRG pain afferents in adults (1, 92). Although we do not have direct evidence that could eliminate the possibility that such functional changes occurred in the present study, NT-3 expression has not been observed in adult GI smooth muscle (50). Therefore, it is unlikely mature vagal GI afferents require NT-3 for normal maintenance or function. This absence of NT-3 expression in mature GI smooth muscle is consistent with our previous observation that NT-3 expression in this tissue began to decrease between E17 and postnatal *day 4* (33, 36). It is also consistent with the more general finding that NT-3 levels decrease with maturation in most tissues that express it, including the intestine (60, 61). Furthermore, although NT-3 expression has been reported in major elastic arteries at adult ages, it is not clear whether the expression we observed during development in the smaller blood vessels that directly supply the GI organs persists into adulthood (33, 36, 75). If this expression does persist, its loss could have hindered the maintenance or function of any vagal sensory axons that reside in close proximity to these blood vessels.

#### Altered development of esophageal IGLEs.

Another possible mechanism that should be addressed is whether the loss of esophageal vagal IGLEs that occurred in global *NT-3* and *trkC* KO mice (71) also occurred in *SM22α-NT-3*^*KO*^ mice. If such loss occurred in these mice, it might have disrupted swallowing, an effect that could have contributed to their reduced eating rate. We suggest this is unlikely for the following reasons. First, during meal pattern testing, group differences in meal duration appeared a couple of days before group feeding rates began to separate (see above). This supports the interpretation that the primary effect of NT-3 loss from smooth muscle was increased meal duration, or reduced satiation, whereas reduced feeding rate was a compensation for this. Second, a mild fast, 6 h during the light phase, normalized (and even slightly, but nonsignificantly increased) the eating rate of *SM22α-NT-3*^*KO*^ mice, an effect unlikely to occur in animals having difficulty swallowing. Finally, NT-3 is not expressed in the outer muscle wall of the esophagus in developing or mature mice, suggesting this tissue cannot be the source of NT-3 that supports development or maintenance of esophageal vagal IGLEs (36, 50). During development, this source is most likely NT-3 produced in the region of the developing nodose ganglion (24).

#### Altered development or function of intrinsic or nonvagal extrinsic innervation of the GI tract.

Another alternative mechanism that could mediate or contribute to the effects of NT-3 loss from GI smooth muscle on feeding is altered development or function of extrinsic and intrinsic neural circuits that regulate digestive reflexes, GI motility in particular. As discussed above regarding vagal mechanoreceptors, however, it is unlikely the mature function of these systems could be directly affected by NT-3 loss from smooth muscle, because NT-3 does not appear to be produced by this tissue in adults. Development of at least some of these systems, however, was likely to have been altered in *SM22α-NT-3*^*KO*^ mice. As described in the Introduction, survival of some vagal afferents is dependent on NT-3, and these could include afferents involved in reflex control of digestive function, as well as those that signal satiation. Furthermore, at least some developing sympathetic and myenteric neurons express trkC, and a significant subpopulation of myenteric neurons is dependent on NT-3 for survival and differentiation (8, 45, 82, 98). In contrast, to our knowledge, potential effects of NT-3 loss on vagal motor (preganglionic) neuron development have not been investigated. These neurons form the final common pathway that mediates vago-vagal reflexes and central nervous system (CNS) control of parasympathetic digestive functions. Although we cannot rule out a subtle effect of the *SM22α-NT-3*^*KO*^ on development of vagal preganglionic neurons in the DMV, it is unlikely they experienced significant disruption. Global KO of *NT-3* appeared to be sensory specific, as all sensory ganglia examined had reduced survival of neurons, whereas motor nuclei were not affected (27, 29, 58). Also, in Nissl-stained brain sections from our *SM22α-NT-3*^*KO*^ mice, the DMV appeared qualitatively normal.

If the decrease in NT-3 expression in the GI tract wall of *SM22α-NT-3*^*KO*^ and *SM22α*^*cre/+*^;*NT-3*^*+/lox*^ mice disrupted development or function of any of these pathways, it may have altered digestive reflexes that could have then indirectly influenced feeding behavior. For example, if GI motility was reduced, then the passage of food through the GI tract would have been retarded. This could have caused animals to eat at a slower-than-normal rate and thus required they extend their meal duration to consume a normal-size meal. The initial feeding patterns of the mice, however, strongly suggested such effects were unlikely to explain the extended meal duration of *SM22α-NT-3*^*KO*^ and *SM22α*^*cre/+*^;*NT-3*^*+/lox*^ mice. On the first day of meal pattern data collection, meal duration was already increased in both of these groups, almost by a factor of two for the *SM22α-NT-3*^*KO*^ mice, whereas the group differences in feeding rate did not begin to appear until *day 3* of testing and did not become consistent until *day 6* onward. This chronology of changes in meal pattern parameters favors the hypothesis that the increased meal duration, rather than decreased eating rate, was the primary effect of the smooth muscle *NT-3* KO and, therefore, that the most direct functional consequence of this manipulation was reduced satiation signaling.

#### SM22α^cre^-mediated recombination in the adult brain of SM22α-NT-3^KO^ mice.

Given the reasonably large literature that established the specificity of the *SM22α* promoter in its targeting of reporter genes and Cre recombinase to smooth muscle (e.g., Ref. 57), it was surprising to find evidence of Cre-mediated recombination in a small number of regions of the adult brain in our laboratory's previous study of smooth muscle KO of *BDNF* (34). In the present study, we also found evidence of recombination in a small number of brain regions, in this instance in the hippocampus, caudomedial entorhinal cortex, and intermediodorsal thalamus of adult *SM22α*^*cre/+*^;*NT-3*^*+/lox-lacZ*^ mice. Although reduced NT-3 in these brain regions could have contributed to the altered feeding behavior of *SM22α-NT-3*^*KO*^ mice, it is unlikely it could account entirely for these effects. Of these three brain regions, only hippocampal manipulations are known to affect food intake. In particular, meal pattern analyses of rodents with hippocampal lesions have observed a reduction in meal size and an increase in meal frequency (13, 17, 32). Moreover, while some studies find no effect of these lesions on food intake and body weight, others have found increases in these measures (13, 17, 32). The medial entorhinal cortex, in conjunction with the interconnected hippocampus, appears to be mainly involved in spatial learning, including that associated with foraging for food (95). This should not have been an issue in the present study, as food was readily available, always from the same location in the home cage, and all mice had adequate time to learn to acquire food from this apparatus before collection of data used for analysis of feeding behavior. Furthermore, to our knowledge, there have not been any reports of manipulations of medial entorhinal cortex or midline thalamic nuclei that altered feeding behavior.

### Evidence Consistent With Involvement of Vagal Afferents That Innervate the GI Tract in Altered Feeding Patterns of SM22α-NT-3^KO^ Mice

#### Comparisons of the effects of SM22α-NT-3^KO^ on feeding behavior with previous studies involving genetic manipulation of vagal GI afferents.

Interestingly, the altered meal patterns of *SM22α-NT-3*^*KO*^ mice were reminiscent of those observed in neurotrophin-4 (*NT-4*) KO mice. *NT-4* KO mice also exhibited an increase in meal duration consistent with reduced satiation, and they partially compensated with a decrease in rate of food consumption (37). These mice had a large loss of vagal IGLE mechanoreceptors from the small intestine, and the remaining IGLEs appeared reduced in size, whereas those innervating the stomach were normal in density, structure, and distribution. Conversely, mice that overexpressed NT-4 appeared to have increased intestinal IGLE innervation and exhibited feeding patterns and responses to cholecystokinin (CCK) consistent with enhanced, rather than reduced, satiation signaling (12). The significant spatial overlap of tissue from which IGLEs density is altered in *NT-4* KO and NT-4 overexpressing mice and the tissue from which NT-3 expression is reduced in *SM22α-NT-3*^*KO*^ mice raises the possibility IGLE density supplying the intestine is reduced by the *SM22α-NT-3*^*KO*^. If this effect occurred, it also could imply NT-4 supports early development of vagal GI afferents, for example, during gangliogenesis (24), whereas, later in development, in particular, upon target innervation, these neurons may switch their neurotrophin requirement to NT-3. Some sympathetic and somatosensory pathways have exhibited such switching of neurotrophin requirements upon reaching their target tissues, in some instances involving NT-3 (7, 26, 31, 38, 54, 70, 91, 98).

#### Comparisons of the effects of SM22α-NT-3^KO^ with previous studies involving surgical or chemical manipulation of vagal GI afferents.

Data derived from studies that utilized chemical and surgical ablation of vagal GI afferents are also consistent with involvement of this innervation in the effects of *SM22α-NT-3*^*KO*^ on feeding behavior. In particular, the present results are consistent with those of previous experiments that have implicated vagally mediated negative feedback signals from the small intestine in short-term regulation of feeding, specifically in the control of meal size, by contributing to meal termination or satiation. The signals include those activated by intestinal load and distension, which are thought to be transduced by mechanoreceptors (77, 90, 96). The most relevant previous studies for comparison are those that utilized methods for disrupting vagal afferents that limited the amount of damage to the vagal motor system (preganglionic neurons). For example, capsaicin damage to a subpopulation of unmyelinated vagal afferents resulted in overconsumption of solid and liquid diets, but only during the initial meal and only with unfamiliar foods (9, 51). In contrast, surgical transection of all vagal GI afferent axons resulted in chronic increases in meal size (liquid diet) due to a combination of increased meal duration and higher maintained lick rates compared with controls (78). The effects of the *SM22α-NT-3*^*KO*^ in the present study, and the similar effects of the *NT-4* KO on meal patterns, fall between these effects of capsaicin and sensory selective vagotomy. Specifically, they resulted in chronic increases in meal duration (solid diet) and a smaller effect on meal size compared with sensory selective vagotomy (liquid diet, *NT-4* KO; Ref. 37). Interestingly, subtle differences in the effects of each type of manipulation on feeding patterns correlate with the degree of deafferentation each one produces. For example, capsaicin reduces a subset of all vagal GI afferents, *NT-4* KO produces selective, but nearly complete loss of intestinal mechanoreceptors and possibly chemoreceptors, and sensory selective vagotomy ablates all vagal GI afferents.

### Perspectives and Significance

Extrinsic innervation of the GI tract is critical for regulation of digestive reflexes and food intake. Sensory input by this innervation is thought to contribute selectively to short-term controls of feeding, whereas long-term controls are thought to be mediated by a CNS regulatory circuit (5). Recent findings, however, have suggested vagal afferents may also play a role in long-term controls (16, 19, 79, 81). The most recent evidence comes from the unexpected finding that plasticity of gut innervation may contribute to diet-induced obesity (DIO). In particular, the long known reduction in CCK-induced satiation in animals with DIO has been shown to result from development of leptin resistance by vagal sensory neurons, which also exhibit reduced excitability and weak activation by CCK and serotonin (15, 16, 19). Importantly, these findings suggested reduced sensitivity of vagal afferents may contribute to DIO, and increasing their sensitivity could help to constrain food intake in DIO.

One strategy to increase vagal afferent sensitivity over the long term would be to first identify key vagal sensory pathways that could be modulated to enhance negative feedback signaling from the gut to the brain to attempt to curtail overeating. This could be achieved by targeted manipulation of neurotrophins or related molecules in specific GI organs or tissues, using an approach similar to that presented here, to increase or decrease the density of functionally distinct subpopulations of vagal GI afferents (33). In fact, our studies of global NT-4 overexpression and smooth muscle-specific *BDNF* KO have shown vagal afferent innervation of specific gut organs contributing to satiation can be increased in density, and that these increases can lead to reduced meal size and increased sensitivity to CCK (6, 12). The contribution of vagal afferent signaling to increased satiation in *SM22α-NT-3*^*KO*^ mice suggested by the present results could imply NT-3 overexpression in smooth muscle might be valuable for decreasing satiation. Additionally, manipulating neurotrophins in peripheral tissues could alter sensitivity of vagal afferents. NT-3 and BDNF are known to modulate sensitivity of primary somatosensory afferents, and BDNF also visceral afferents (20, 92).

A second potential application for gut-targeted neurotrophin models may be in manipulating plasticity of vagal afferents in obesity. Plasticity of BDNF and its receptor, trkB, have been observed in the hypothalamus, hippocampus, and vagal sensory neurons in animals with DIO (64, 97), raising the possibility that NT-3 and BDNF produced by mature GI tissues could exhibit similar plasticity (50, 59). Indeed, evidence suggests neurotrophins are involved in plasticity of visceral sensory systems (67). Additionally, some of the cellular signaling molecules activated by neurotrophins overlap with those mediating effects of leptin and CCK on vagal afferent plasticity, including protein kinase C, cyclic AMP response element binding protein, and Egr-1 (cf. Refs. 21, 47). Plasticity of BDNF or trkB in vagal sensory neurons could lead to reduced vagal afferent sensitivity to neurotransmitters or hormones that promote satiation (93). Consistent with this possibility, vagal sensory neurons exhibit increased BDNF expression in hypertensive rat models, an effect that may play a role in plasticity associated with adaptive responses of vagal cardiovascular afferents (89). The genetic targeting strategy utilized in the present study could be applied to test whether neurotrophins do in fact play a role in vagal GI afferent plasticity associated with DIO and whether preventing these effects can reduce DIO.

The results of these types of experiments utilizing gut-targeted manipulation of neurotrophins or related molecules are likely to identify pathways involved in the sensory feedback regulation of eating behavior that could be targeted to treat obesity. A major advantage of such a treatment strategy, focused on peripheral nervous system modulation, would be the possibility of reduced side effect potential compared with treatments directed to the CNS.

## GRANTS

This research was supported by National Institute of Neurological Disorders and Stroke Grant R01 NS-046716 to E. A. Fox, by a Burroughs-Wellcome New Investigator in Pharmacology Award to K. R. Jones, and by a Howard Hughes Medical Institute Fellowship to Z. C. Baquet.

## DISCLOSURES

No conflicts of interest, financial or otherwise, are declared by the author(s).

## AUTHOR CONTRIBUTIONS

Author contributions: E.A.F. conception and design of research; E.A.F., J.E.B., Z.C.B., and J.M. performed experiments; E.A.F. and J.E.B. analyzed data; E.A.F. interpreted results of experiments; E.A.F. and K.R.J. prepared figures; E.A.F. drafted manuscript; E.A.F., J.E.B., and K.R.J. edited and revised manuscript; E.A.F., J.E.B., Z.C.B., K.R.J., and J.M. approved final version of manuscript.
